# Quercetin Ameliorates Gut Microbiota Dysbiosis That Drives Hypothalamic Damage and Hepatic Lipogenesis in Monosodium Glutamate-Induced Abdominal Obesity

**DOI:** 10.3389/fnut.2021.671353

**Published:** 2021-04-29

**Authors:** Lijun Zhao, Xiaoqiang Zhu, Mengxuan Xia, Jing Li, An-Yuan Guo, Yanhong Zhu, Xiangliang Yang

**Affiliations:** ^1^National Engineering Research Center for Nanomedicine, College of Life Science and Technology, Huazhong University of Science and Technology, Wuhan, China; ^2^Key Laboratory of Molecular Biophysics of the Ministry of Education, Department of Bioinformatics and Systems Biology, College of Life Science and Technology, Huazhong University of Science and Technology, Wuhan, China

**Keywords:** abdominal obesity, gut microbiota, retinol saturase, hypothalamic damage, dietary quercetin

## Abstract

Monosodium glutamate (MSG)-induced abdominal obesity, conventionally caused by hypothalamic damage, is a critical risk factor for health problem. Microbiota-gut-brain axis plays important roles in a variety of metabolic diseases. However, whether gut microbiota is involved in the pathogenesis for MSG-induced abdominal obesity and the effect of quercetin on it remains unclear. Herein, we find that MSG-induced gut microbiota dysbiosis contributes to neuronal damage in the hypothalamus, as indicated by antibiotics-induced microbiota depletion and co-house treatment. Inspired by this finding, we investigate the mechanism in-depth for MSG-induced abdominal obesity. Liver transcriptome profiling shows retinol metabolism disorder in MSG-induced abdominal obese mice. In which, retinol saturase (RetSat) in the liver is notably up-regulated, and the downstream lipogenesis is correspondingly elevated. Importantly, microbiota depletion or co-house treatment eliminates the difference of RetSat expression in the liver, indicating gut microbiota changes are responsible for liver retinol metabolism disorder. Moreover, this study finds dietary quercetin could modulate MSG-induced gut microbiota dysbiosis, alleviate hypothalamic damage and down-regulate liver RetSat expression, thus ameliorating abdominal obesity. Our study enriches the pathogenesis of MSG-induced abdominal obesity and provides a prebiotic agent to ameliorate abdominal obesity.

## Introduction

Abdominal obesity is a critical risk factor in the development of cardiovascular and metabolic syndromes independently of overall adiposity (1, 2). The prevalence of abdominal obesity is showing rapid growth over several decades (3, 4). Monosodium glutamate (MSG), an umami substance, plays a crucial role in inducing abdominal obesity (5). Conventionally, MSG-induced abdominal obesity is attributed to neuronal damage in the mouse hypothalamus. Olney et al. proved that hypothalamic damage reduced pituitary function to promote abdominal obesity after subcutaneous injected with MSG (6). Hermanussen et al. believed that MSG intake could damage the appetite to induce abdominal obesity (7). In recent years, it was found that alternations of obesity-associated gut microbial species were correlated with serum glutamate concentration, which can be considered as a potential biomarker of abdominal obesity (8, 9). Microbiota-gut-brain axis, a bidirectional communication system between the gastrointestinal tract and brain, affects the development of a variety of complex metabolic diseases (10). However, how gut microbiota changes and interacts with hypothalamus function and other metabolic alterations in the pathogenesis of MSG-induced abdominal obesity remains unknown.

Drugs and dietary supplements selected from the existing substances to effectively treat MSG induced-abdominal obesity is desired. Quercetin, a polyphenolic flavonoid extracted from sea buckthorn, hawthorn, onion, etc., has shown to be a fat-lowering molecule due to its function in lipolysis, fatty acid uptake and inhibition of lipogenesis (11, 12). Studies proved that quercetin could significantly reduce total body fat, waist circumference and body mass index of obese subjects (13, 14). However, it is unclear whether quercetin can affect the microbiota and brain to ameliorate abdominal obesity.

In this study, fecal 16S ribosomal DNA (rDNA) sequencing analysis shows obvious gut microbiota dysbiosis in MSG-induced abdominal obesity, which contributes to hypothalamic damage, as demonstrated by gut microbiota depletion and co-house treatment. What' more, we disclose that such gut microbial changes up-regulate liver retinol saturase (RetSat) expression by liver transcriptomics profiling, which results in evaluated lipid accumulation. Moreover, quercetin treatment could improve MSG-induced abdominal obesity by restoring gut microbiota dysbiosis-mediated hypothalamus damage and retinol metabolism disorder. Our study enriches the pathogenesis of MSG-induced abdominal obesity and provides a reference basis for drug treatment of abdominal obesity.

## Materials and Methods

### Animals

All C57BL/6J mice were purchased from Hubei Center for Disease Control and Prevention, China [quality certification number: SCXK (E) 2015-0018]. The mice were maintained in cages at 22 ± 2°C, 55 ± 5% relative humidity and with a 12 h light-dark cycle. The animal studies were approved by the Institutional Animal Care and Use Committee at Tongji Medical College, Huazhong University of Science and Technology (IACUC Number: 2170). The abdominal obesity model was prepared according to the literature (15). Briefly, the neonatal mice were subcutaneously injected with saline or MSG (3 mg/g body weight, dissolved in saline, Sinopharm Chemical Reagent Co., Ltd.) from day 2 to 8 once daily. After weaning, the male mice had access to chow food and water *ad libitum*. Body weight and food intake of the two groups were recorded weekly. The body lengths were determined by measuring nasal-to-anal distance. Lee's index was determined by applying a formula [(3 square root body weight (g))/body length (mm)×10] (16). At week 18, all mice were sacrificed; brain, liver, adipose and colon tissues were removed and stored at −80°C.

### Metabolic Activity

To monitor metabolic activities, mice were kept individually in metabolic cages (Comprehensive Lab Animal Monitoring System, Columbus, USA). Oxygen consumption, food intake, total locomotor activity accounts (determined as the interrupted numbers of infrared light beam), respiratory exchange ratio (VCO_2_ /VO_2_), and heat production were measured during the 48 h.

### 16S rDNA Sequencing

Feces collected from mice at week 18 were immediately frozen in liquid nitrogen and stored at −80°C. Fecal microbial DNA was isolated by the Omega Bio-tek stool DNA kit (Omega, Norcross, GA, USA) and quantified by NanoDrop 2000 spectrophotometer (Thermo Scientific, USA). DNA samples were quantified, followed by amplification of V4 hypervariable region of the 16S rDNA. Final amplicon pool was evaluated by the AxyPrep DNA gel extraction kit. Paired-end reads were generated with Illumina MiSeq PE250 (Beijing Genomics Institute, Shenzhen, China), and the reads were filtered out with default parameters. The tags were clustered into OTU with a 97% threshold by using UPARSE, and the OTU unique representative sequences were obtained; Chimeras were filtered out by using UCHIME (v4.2.40) (17). The data in different samples were summarized in a profiling table or histogram, and the histogram was drawn using the software R (v3.1.1).

### mRNA Sequencing and Differential Expression Analysis

Total RNAs from liver tissues were extracted using TRIzol reagent (Invitrogen Life Technologies, Rochester, NY, United States). Sequencing libraries were generated using NEBNext Ultra RNA Library Prep Kit for Illumina (NEB, USA) following manufacturer's recommendations and index codes were added to attribute sequences to each sample. The library preparations were sequenced on an Illumina Hiseq 4000 platform and 150bp paired-end reads were generated (Novogene, Beijing, China). Low quality reads were removed by in-house scripts. The following reads were discarded: (1) reads < 35 bp after adapter trimming; (2) reads with multiple N (>5 bases); (3) reads with low quality bases (quality value ≤ 5, ratio of low quality bases > 10%). RNA-seq reads were aligned by hisat2 (version 2.05), and Cufflinks (v2.1.1) was used to estimate the expression of genes. The NOISeq package was used to conduct the differentially expression of genes (DEGs) (prob >0.9 and |fold change| > 2) (18). Then, enrichment analysis of DEGs was performed by the DAVID Functional Annotation Tool (https://david.ncifcrf.gov/). The MeV (http://mev.tm4.org) software with hierarchical clustering method was used to present the expression heatmap, showing the expression of genes in Retinol metabolism pathways.

### Antibiotic Treatment

To deplete gut microbiota, the mice were given antibiotics (ABX) (vancomycin 0.5 mg/mL, ampicillin 1 mg/mL, neomycin 1 mg/mL, and metronidazole 1 mg/mL; Sigma, Sangon Biotech, China) in drinking water *ad libitum* after birth (19). Antibiotic treatment was continued for 18 weeks until sacrifice. All mice were fed on chow diet.

### Co-housing Experiment

The neonatal mice were subcutaneously injected with saline or MSG as above described. After weaning, the male mice were divided into three groups: control group, MSG group and Co-house group (the mice injected with MSG were co-housed with control mice for 18 weeks). The mice had free access to food and water.

### Quercetin Treatment

The abdominal obesity model was constructed as described in 2.1. After 3 months, the MSG-treated mice were randomly divided into two groups: MSG group and quercetin group (Que, 5 mg/kg quercetin, dissolved in 0.15% carboxymethylcellulose sodium, Push Bio-technology). Quercetin was administrated by gavage at a dose of 100 μL/10 g body weight once per day for 6 weeks.

### Biochemical Analysis

Blood samples were collected for separating sera. The liver retinol and retinol saturase content were determined by enzyme-linked immunosorbent assay (ELISA) according to the instruction (69-58263, 69-35272, MSKBIO, China). All samples were measured in triplicate.

### Histology and Immunohistochemistry

Tissues were fixed in 4% paraformaldehyde, embedded in paraffin, and processed for histological analysis. Liver, brain, adipose and colon tissues were stained with hematoxylin and eosin (H&E) for general morphological observations. And liver tissues were also stained with Oil Red O. Colon tissues were performed with periodic acid-schiff (PAS) staining to determine the content of mucin protein. The images were acquired using a light microscope (Nikon Eclipse TE2000-U, NIKON, Japan). Damaged neurons were indicated by the increased eosinophilic neurons in the arcuate nucleus. The morphometric analyses of tissues were made by the software Image Pro Plus 5.0 (Media Cybernetics, USA).

### Quantitative Real-Time PCR

Liver and colon tissues were homogenized in RNAiso Plus reagent. Centrifugation was performed after chloroform was added at 12,000 rpm for 15 min (4°C). The supernatant was collected and precipitated with isopropanol for 30 min at room temperature. Then the RNA pellet was obtained by centrifuging at 12,000 rpm for 10 min (4°C), and washed in 75% ethanol and then centrifuged at 7,500 rpm for 5 min (4°C). After resuspended in DEPC water, RNA was quantified with Nanodrop 2000 spectrophotometer (ThermoScientific, USA). The cDNA was synthesized with the PrimeScript RT reagent Kit and amplified using 7500 Real Time PCR System (Applied Biosystems, USA). Expression levels were calculated using the cycle threshold (CT) comparative method (2^−*ddCT*^) normalizing to *Actin* CT values. The primer sequence was shown in Supplementary Table 1.

### Cell Culture

MSG-induced obese mice were anesthetized by intraperitoneal injection of pentobarbital sodium and livers were perfused via the inferior vena cava with 1 mL PBS and 30 mL digestion buffer (collagenase IV, Gibco). The excised liver was scraped, the suspension filtered through a 70 μm mesh filter, and hepatocytes were resuspended by 90% Percoll Plus solution (GE Healthcare). Then hepatocytes were incubated in Dulbecco's modified Eagle's medium (DMEM), 10% calf serum (CS) and 1% penicillin and streptomycin (Gibco). The hepatocytes were collected by centrifugation at 1,200 rpm for 3 min, and resuspended in opti-MEM at a concentration of 4 × 10^5^/mL. Hundred microliter hepatocytes were transfected with 20 μM siRNA (GenePharma) (Supplementary Table 1) and 5 μL GP-siRNA-Mate Plus (GenePharma), respectively, at room temperature for 20 min, and then transferred into 6-well plate containing 1.8 mL opti-MEM for culture. After 6 h, the cell viability was tested by CCK8 and the culture medium was replaced by DMEM with 10% CS and 1% penicillin and streptomycin.

### Statistical Analysis

Data are expressed as mean and standard error of mean (SEM). Statistical analysis was performed with GraphPad Prism software and the SPSS 20 software (SPSS Inc., Chicago, USA). The statistical differences between groups were evaluated by one-way ANOVA or two-way ANOVA followed by *post-hoc* LSD tests. In any case, *p* < 0.05 was considered as statistically significant.

## Results

### Microbiota Depletion Attenuates MSG-Induced Hypothalamic Damage and Abdominal Obesity

**Figure 1:**
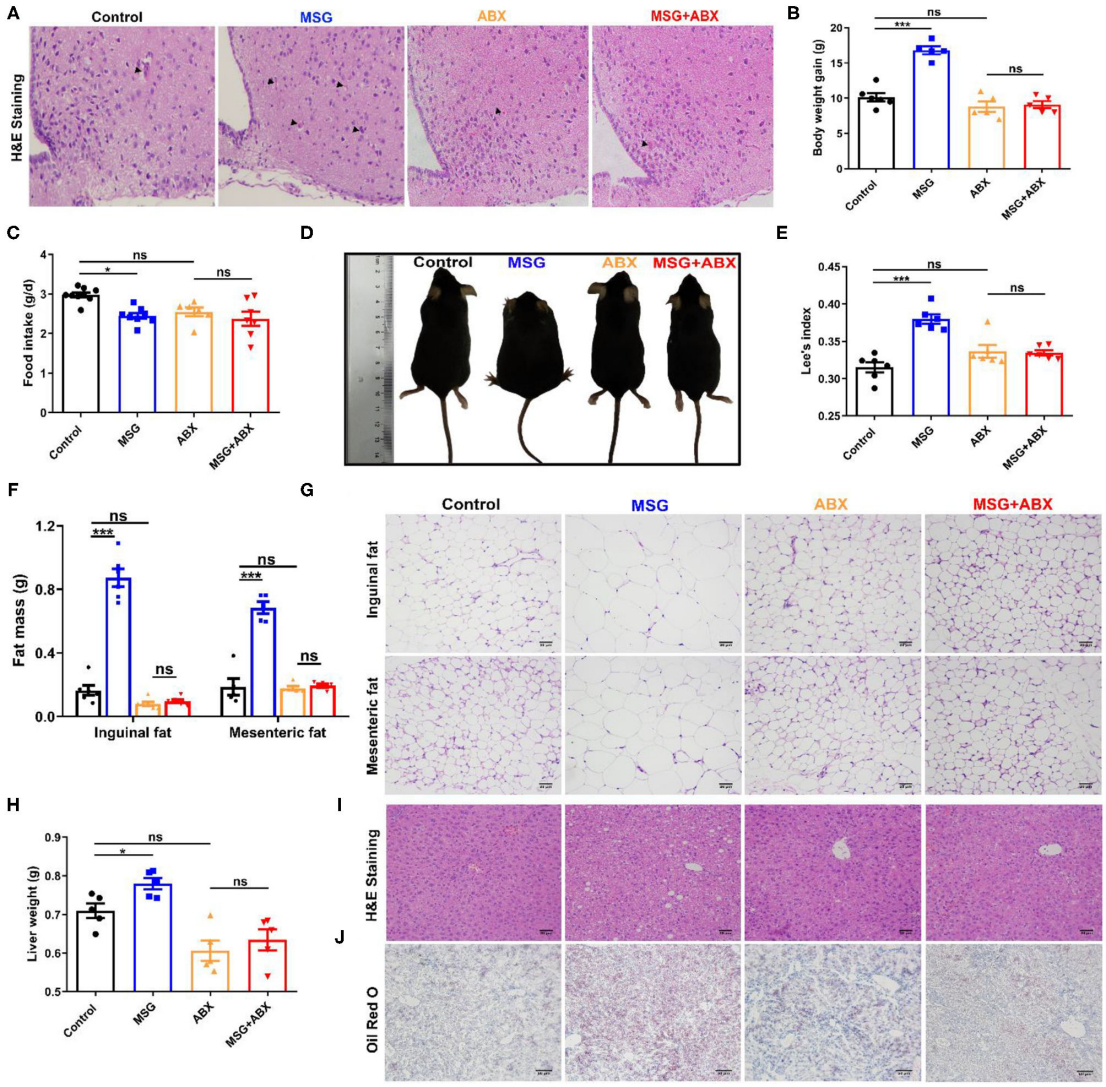
MSG-induced abdominal obesity is counteracted by ABX treatment. **(A)** H&E staining in the arcuate nucleus of hypothalamus. **(B)** Body weight gain. **(C)** Food intake per day. **(D)** Mice morphology. **(E)** Lee's index. **(F)** Fat mass and **(G)** H&E staining of inguinal and mesenteric adipose tissues. Scale bar: 20 μm. **(H)** Liver weight. **(I)** H&E staining of liver tissues. Scale bar: 20 μm. **(J)** Oil Red O staining of liver tissues. Scale bar: 50 μm. Data are presented as mean ± SEM, *n* = 5–6. Data were analyzed by two-way ANOVA followed by *post-hoc* LSD tests. *p < 0.05, ***p < 0.001, ns, no significance. Arrowheads indicate eosinophilic neurons.

MSG-induced abdominal obesity was correlated to neuronal damage in the hypothalamus (6), which was confirmed by the increased eosinophilic neurons (arrowhead indicated) in the MSG-treated mice (Figure 1A). As expected, the MSG-treated mice displayed a significant increase in the body weight gain (Figure 1B), whereas the food intake was lower than that in control mice (Figure 1C). In MSG-treated mice, shorter body length and increased Lee's index, an index of MSG-induced abdominal obesity (16), were manifested compared with those in control mice (Figures 1D,E). In line with body weight gain, the MSG-treated mice showed significantly higher inguinal and mesenteric fat mass (Figure 1F). H&E staining results indicated an increase of adipocyte size in inguinal and mesenteric adipose tissues of the MSG-treated group (Figure 1G, Supplementary Figure 1). Besides, we found that liver weight was notably increased in the MSG-treated mice (Figure 1H), which may be attributed to more lipid droplets accumulation in liver tissues determined by H&E and Oil Red O staining (Figures 1I,J). To monitor basic metabolic indicators, all mice were kept in metabolic cages. It was found that oxygen consumption, respiratory exchange ratio, heat production and locomotor activity was notably reduced in the MSG-treated mice (Supplementary Figure 2), indicating that the MSG-treated mice had lower metabolic levels. These results elucidated that MSG administration could induce hypothalamic damage and abdominal obesity.

Interestingly, after gut microbiota depletion by the ABX treatment (Supplementary Figure 3A), the microbiota community in control and abdominal obese mice became similar (Supplementary Figure 3B). And severe neuronal damages were disappeared in the MSG-treated group (Figure 1A), indicating that gut microbiota might be involved in MSG-induced neuronal damage. Importantly, the results showed that there was no obvious difference in body weight gain (Figure 1B), as well as in body length and Lee's index between control and MSG mice after ABX treatment (Figures 1D,E). And differences in liver lipid deposition, adipocyte size and metabolic level disappeared (Figures 1F–J, Supplementary Figures 1, 2). To further confirm the role of gut microbiota in abdominal obesity, the MSG-treated mice and the control mice were co-housed after weaning. As we expected, the neuronal damage and abdominal obesity characteristics were ameliorated by the co-house treatment (Supplementary Figure 4). These results demonstrated that the intestinal flora played crucial roles in the development of MSG-induced neuronal damage and abdominal obesity.

### MSG Leads to Markable Gut Microbiota Dysbiosis

**Figure 2:**
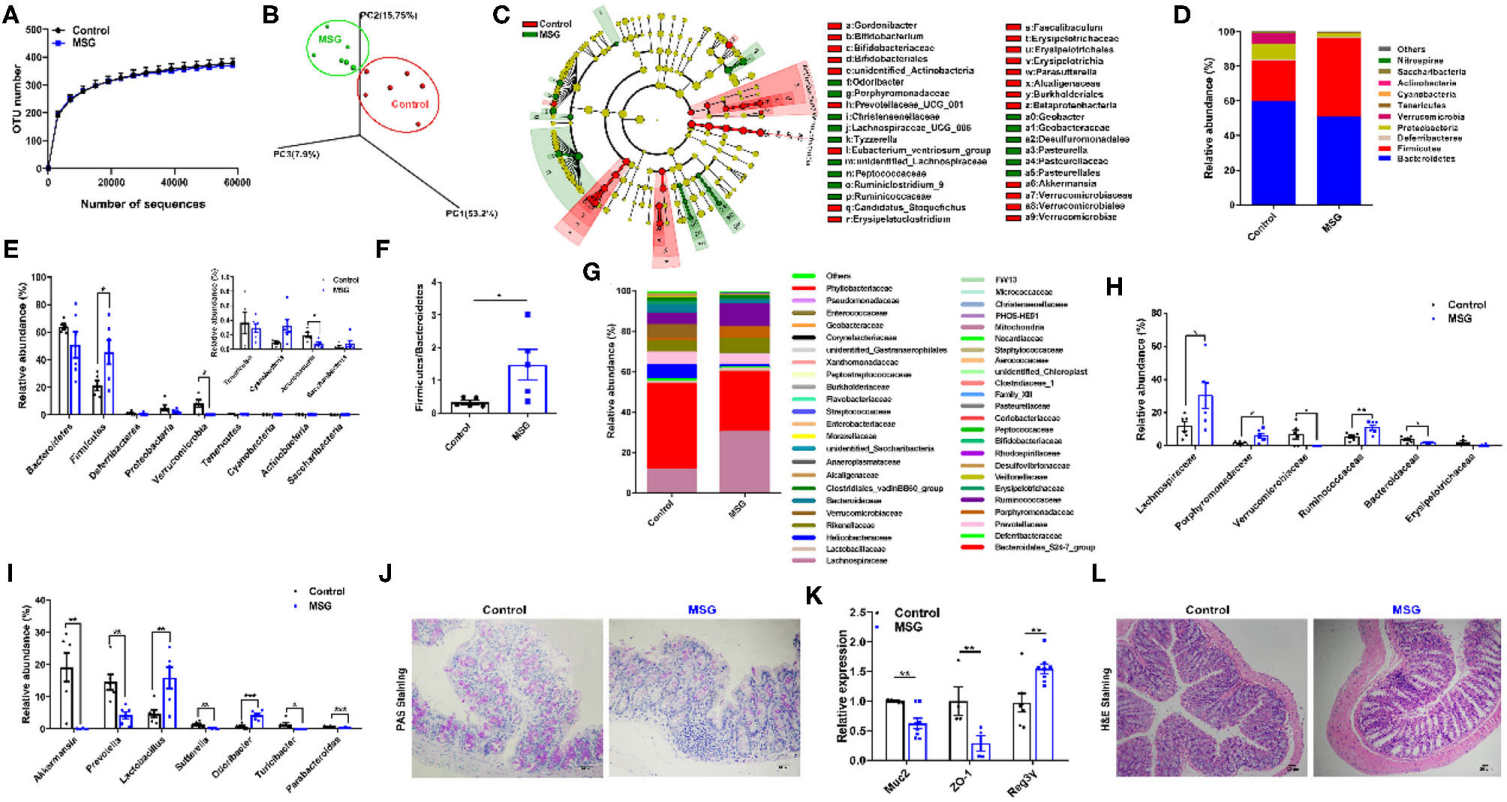
MSG administration induces gut microbiota dysbiosis and damaged gut barrier. **(A)** OTU number accumulation analysis of 16S rDNA sequencing. **(B)** Weighted Unifrac analysis of microbiota in the control and MSG group. **(C)** Linear discriminant analysis effect size (LEfSe) of microbiota in the control and MSG group. **(D)** Microbiota composition and **(E)** relative abundances of microbiota at the phylum level. **(F)** The ratio of *Firmicutes* to *Bacteroidetes*. **(G)** Microbiota composition and **(H)** relative abundances of microbiota at the family level. **(I)** Relative abundance of flora at the genus level. **(J)** Periodic acid-schiff (PAS) staining of colon tissue. Scale bar: 20 μm. **(K)** The gene expression of *Muc2, ZO-1*, and *Reg3*γ in colon. **(L)** H&E staining of colon tissue. Scale bar: 20 μm. Data are presented as mean ± SEM, *n* = 6. Data were analyzed by one-way ANOVA followed by *post-hoc* LSD tests. *p < 0.05, **p < 0.01, ***p < 0.001.

Subsequently, in order to investigate the changes in the intestinal flora, fecal 16S rDNA sequencing was conducted. The α-diversity analysis showed that the operational taxonomic units (OTU) number had no difference between the control group and MSG-treated group (Figure 2A). However, weighted Unifrac analysis and linear discriminant analysis effect size (LEfSe) revealed significant different microbiota compositions, indicating the dramatically changes in gut microbial profile after MSG administration (Figures 2B,C). Then we examined the changes at the phylum level and found that the abundance of *Firmicutes* was significantly higher, whereas the abundance of *Verrucomicrobia* was notably lower in the MSG-treated group compared with the control group (Figures 2D,E). And the *Firmicutes*/*Bacteroidetes* ratio, a hallmark related to obesity (20), was remarkably increased in the MSG-treated group (Figure 2F). At the family level, the abundance of *Lachnospiraceae* and *Ruminococcaceae*, which have been proven to induce diabetes and obesity (21), was also significantly higher (Figures 2G,H). The abundance of *Bacteroidaceae*, which could effectively use polysaccharides to produce anti-inflammation short-chain fatty acids (SCFAs) (22), was lower in the MSG-treated group (Figures 2G,H). At the genus level, the abundance of *Akkermansia* was significantly decreased in the MSG-treated mice (Figure 2I). As a kind of anaerobic bacterium living in the mucus layer, *Akkermansia* improves gut barrier function and inversely correlates with body weight (23). We speculated the mucus layer may be affected by *Akkermansia* in the MSG-treated mice. As expected, periodic acid-schiff (PAS) staining results showed intestinal mucus secretion decreased (Figure 2J), which was further verified by down-regulation of *Muc2* determined by qRT-PCR in the MSG-treated group (Figure 2K). Besides, the decreased level of *ZO-1* gene encoding tight junction protein-1, and the increased level of antimicrobial peptide *Reg3*γ indicated the gut barrier was damaged. Moreover, inflammation filtration was found in damaged colon tissues by H&E staining (Figure 2L). Therefore, MSG administration resulted in greatly altered gut microbiome and damaged gut barrier function in abdominal obese mice.

It was worth mentioning that shorter colon length, increased inflammation infiltration and reduced mucus secretion in MSG-induced mice were reversed after ABX or co-house treatments (Supplementary Figures 5, 6), indicating the importance of gut microbiota alterations in the intestinal barrier function in MSG-induced abdominal obesity.

### Gut Microbiota Affects RetSat Level in the Liver

**Figure 3:**
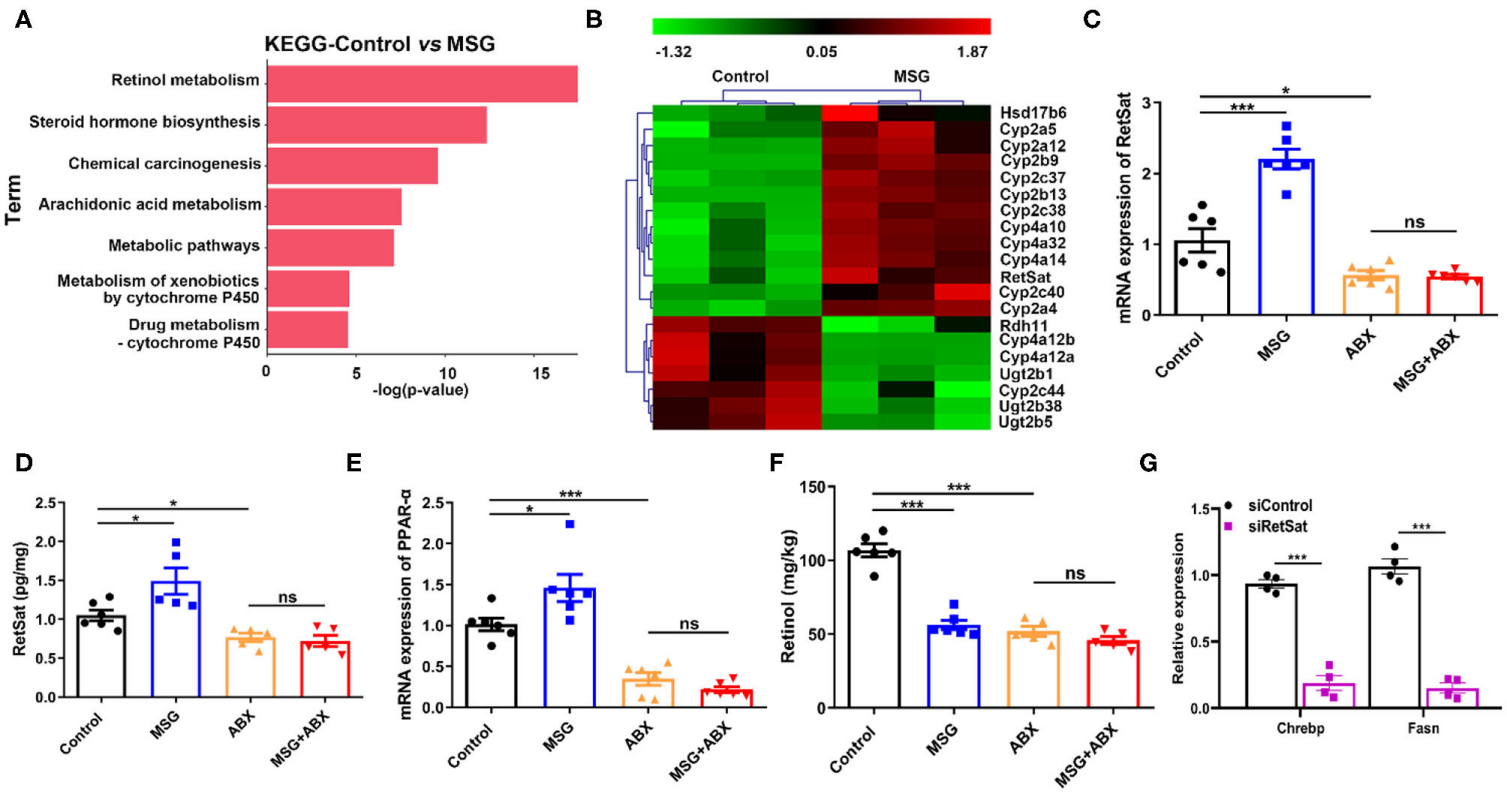
Liver RetSat level is affected by gut microbiota dysbiosis. **(A)** The functional enrichment of DEGs in the control and MSG group (*n* = 3). **(B)** Heatmap representing the genes significant different in the control and MSG group (*p* < 0.05). **(C)** mRNA expression tested by qRT-PCR **(D)** and protein content tested by ELISA of liver RetSat. **(E)** mRNA expression of PPAR-α in the liver. **(F)** Retinol content in the liver. **(G)** mRNA expression of *Chrebp* and *Fasn* in siControl or siRetSat-treated hepatocytes by qRT-PCR. Data are presented as mean ± SEM, *n* = 4–6. Data were analyzed by two-way ANOVA followed by *post-hoc* LSD tests. *p < 0.05, ***p < 0.001, ns, no significance.

Emerging evidence suggested that the interactions between the liver and the gut microbiota play crucial roles in chronic diseases (24). We wondered whether microbiota changes induced by MSG affect the liver metabolism. Transcriptome profiling analysis showed that the differentially expressed genes (DEGs) in abdominal obese mice were mainly enriched in pathways of “Retinol metabolism pathway,” “Steroid hormone biosynthesis” and “Chemical carcinogenesis” (Figure 3A). In which, retinol metabolism was greatly affected. It is reported that retinol metabolism signaling can regulate pathways involved in obesity, such as hepatic lipid metabolism and inflammation pathway (25, 26). Among genes related to retinol metabolism, we noticed retinol saturase (RetSat), which catalyzes the retinol to 13,14-dihydroretinol, was reported to coordinate liver lipid metabolism by upregulating Chrebp activity related to lipogenesis (27), was markedly increased in the MSG-treated mice (Figure 3B), which was also confirmed by qRT-PCR and ELISA assay (Figures 3C,D). PPAR-α (peroxisome proliferator-activated receptor-a), a nuclear transcriptional factor mediating RetSat expression in the liver (28), was significantly increased in the MSG-treated mice (Figure 3E). Moreover, the levels of *Cyp4a10, Cyp4a32*, and *Cyp4a14*, which regulated PPAR-α transcriptional activity (29), were also upregulated in the transcriptome analysis (Figure 3B). Figure 3F showed that the retinol, the substrate of RetSat, was decreased along with the increased expression of RetSat in the MSG-treated mice by ELISA assay, which confirmed the elevation of RetSat activity in abdominal obese mice. To further certify its function, we silenced the RetSat expression of hepatocytes in the MSG-induced obese mice by siRNA. After silence of RetSat (Supplementary Figure 7), *Chrebp* and *Fasn*, genes related to lipogenesis, were notably downregulated (Figure 3G), which was consistent with the previous report (27).

Intriguingly, *RetSat* levels between control and MSG mice were found no difference after microbiota depletion (Figures 3C,D), indicating that gut microbiota could regulate RetSat expression in the liver. Besides, we found *PPAR-*α and retinol showed no differences between the control and MSG group after microbiota depletion (Figures 3E,F), which was coincided with the study that gut microbiota could regulate liver PPAR-α expression (30). Taken together, we demonstrated that MSG-induced gut microbiota changes increased the RetSat expression, which further promoted lipid synthesis in liver.

### Quercetin Alleviates MSG-Induced Hypothalamic Damage and Abdominal Obesity

**Figure 4:**
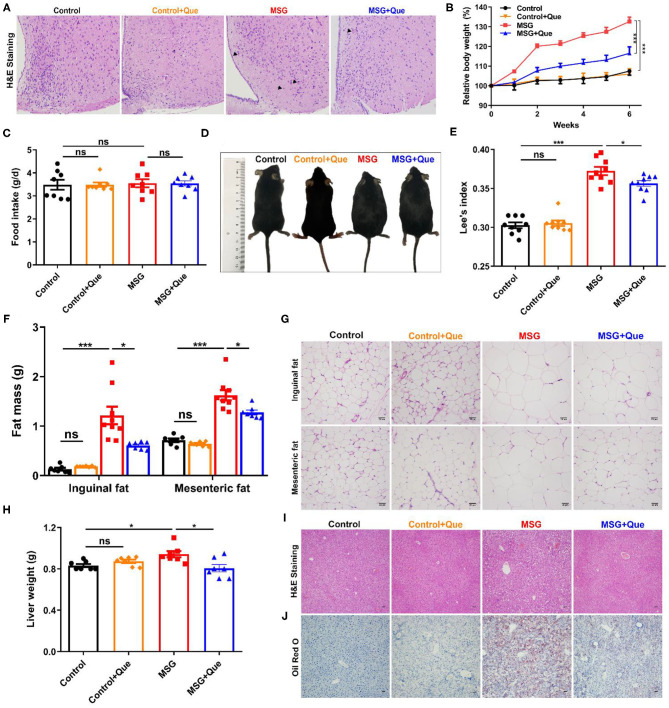
Quercetin treatment alleviates MSG-induced hypothalamic damage and abdominal obesity. **(A)** H&E staining in the arcuate nucleus of hypothalamus. **(B)** Relative body weight. **(C)** Food intake per day. **(D)** Mice morphology. **(E)** Lee's index. **(F)** Fat mass and **(G)** H&E staining of inguinal and mesenteric adipose tissues. Scale bar: 20 μm. **(H)** Liver weight. **(I)** H&E staining and **(J)** Oil Red O staining of liver tissues. Scale bar: 20 μm. Data are presented as mean ± SEM, *n* = 5–10. Data were analyzed by two-way ANOVA followed by *post-hoc* LSD tests. *p < 0.05, ***p < 0.001, ns, no significance. Arrowheads indicate eosinophilic neurons.

As abdominal obesity is serious for cardiovascular and metabolic syndrome (1, 2, 31), developing medicines for its prevention is urgent. Quercetin, previously described to exert anti-inflammation and anti-obesity effects (32, 33), might be a potential candidate for improving metabolic disorders. Firstly, we proved that quercetin had no influence on body weight gain, food intake, waist circumference, Lee's index, liver weight, adipose tissue mass and metabolic levels in normal mice (Figure 4, Supplementary Figures 8, 9). Then, quercetin was orally administrated to investigate its effects on abdominal obesity. The results showed that increased eosinophilic neurons (arrowhead indicated) in the MSG-treated group were reversed after quercetin treatment (Figure 4A). The body weight of MSG-treated mice was significantly decreased after the quercetin treatment (Figure 4B), along with no difference in food intake between the two groups (Figure 4C). Correspondingly, waist circumference and Lee's index were clearly reduced after quercetin treatment (Figures 4D,E). In accordance with body weight loss, quercetin treatment significantly reduced inguinal and epididymal fat mass in MSG-treated mice (Figure 4F). Consistent with the above results, H&E staining results showed that adipocyte sizes of epididymal and inguinal adipose tissues in the quercetin-treated group was reduced significantly (Figure 4G, Supplementary Figure 8). The liver weight was reduced (Figure 4H), and MSG-induced hepatic lipid deposition was also alleviated after quercetin treatment (Figures 4I,J). In addition, metabolic cage experiment results showed that respiratory exchange ratio, which was used to determine the portion of carbohydrates and fats utilized (34), was lower in quercetin-treated mice (Supplementary Figure 9), indicating increased lipid oxidation after quercetin treatment. Oxygen consumption, heat production and locomotor activity were notably increased in MSG-induced obese mice after quercetin treatment (Supplementary Figure 9), indicating higher metabolic levels. Therefore, our results demonstrated that quercetin treatment could alleviate brain neuronal damage and MSG-induced abdominal obesity.

### Quercetin Improves MSG-Induced Microbiota Dysbiosis and Gut Barrier Function

**Figure 5:**
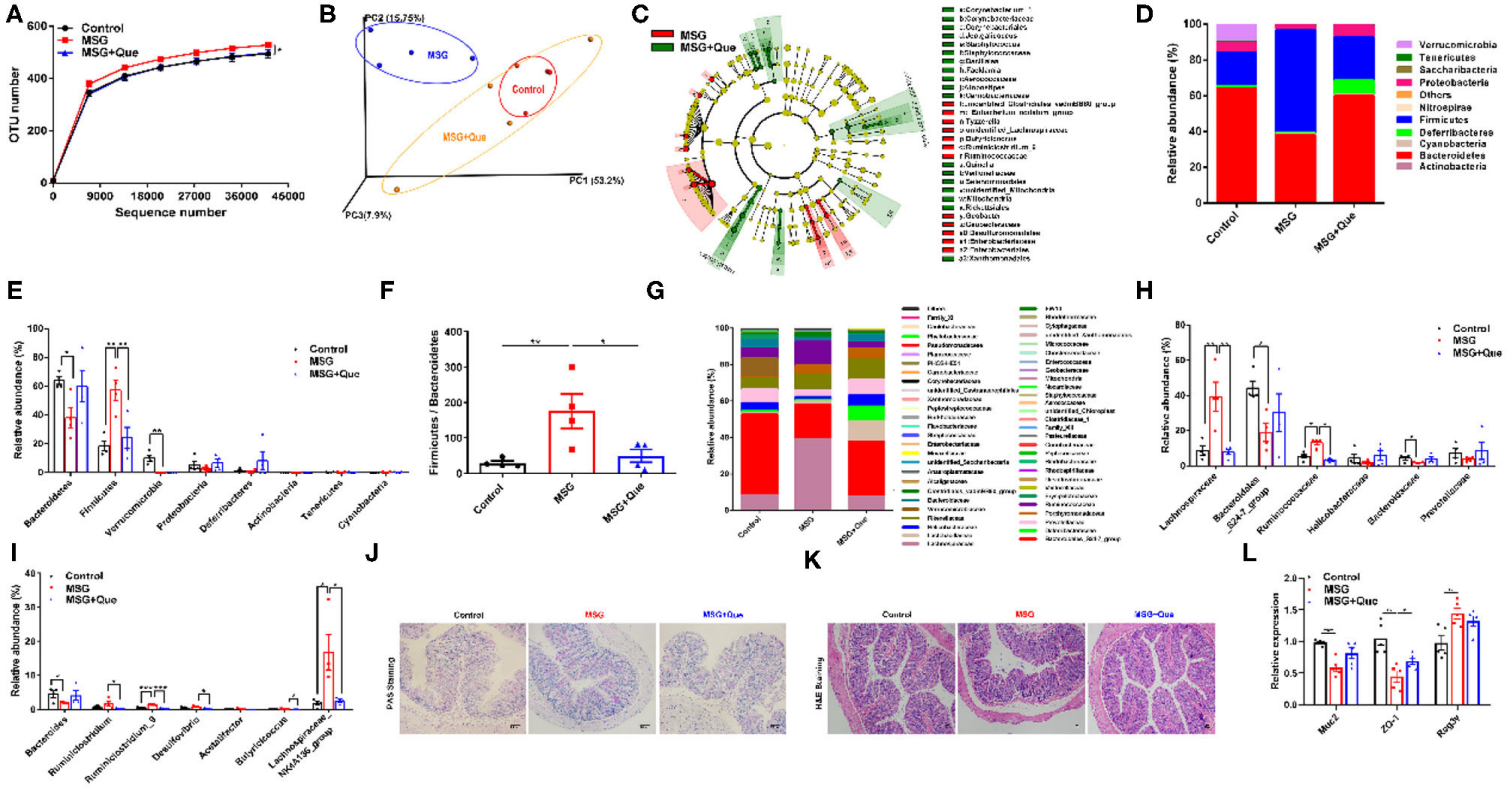
MSG-driven microbiota dysbiosis and damaged gut barrier is attenuated after the quercetin treatment. **(A)** OTU number and **(B)** Weighted Unifrac analysis of the control, MSG and MSG+Que group. **(C)** LEfSe analysis of microbiota in the MSG and MSG+Que group. **(D)** Microbiota composition and **(E)** relative abundance of gut flora at the phylum level. **(F)** The ratio of *Firmicutes* to *Bacteroidetes*. **(G)** Microbiota composition and **(H)** relative abundance of gut microbiota at the family level. **(I)** Relative abundance of flora at the genus level. **(J)** PAS staining and **(K)** H&E staining of colon tissue. Scale bar: 20 μm. **(L)** The gene expression of *Muc2, ZO-1*, and *Reg3*γ in colon tissues. Data are presented as mean ± SEM, *n* = 4–6. Data were analyzed by one-way ANOVA followed by *post-hoc* LSD tests. *p < 0.05, **p < 0.01, ***p < 0.001.

As described above, we demonstrated that MSG-induced abdominal obesity was affected by gut microbiota-mediated liver RetSat expression. Due to the modulation of polyphenols on gut microbiota (35), we speculate that the mechanism of quercetin in improving abdominal obesity is to ameliorate gut microbiota dysbiosis. The gut microbiota analysis indicated that the OTU number was declined after the quercetin treatment compared with that in the MSG-treated group (Figure 5A). Weighted Unifrac analysis results revealed the quercetin treatment could ameliorate MSG-induced gut microbial dysbiosis, making the microbiota structure more similar to that in control mice (Figure 5B). LEfSe analysis revealed the gut microbiota alterations in the MSG group was reversed by the quercetin treatment (Figure 5C). Specifically, the elevated abundance of *Firmicutes* was reversed after the quercetin treatment (Figures 5D,E). Moreover, *Firmicutes*/*Bacteroidetes* ratio was dramatically decreased after the quercetin treatment (Figure 5F). Then we explored the changes at the family level and found that the increased abundance of *Lachnospiraceae* and *Ruminicoccaceae* in the MSG-treated group was effectively reversed after the quercetin treatment (Figures 5G,H). The results showed the decreased abundance of *Bacteroides* at the genus level in the MSG-treated group was increased after the quercetin treatment (Figure 5I). Besides, the damaged colon tissues and disrupted mucus secretion in the MSG-treated group were recovered after the quercetin treatment (Figures 5J,K). The upregulated expression of *Muc2* and *ZO-1*, and downregulated expression of *Reg3*γ were found after the quercetin treatment (Figure 5L). These results proved quercetin could improve gut microbiota dysbiosis and intestinal barrier function in abdominal obesity.

### Quercetin Down-Regulates Liver RetSat in MSG-Induced Obese Mice

**Figure 6:**
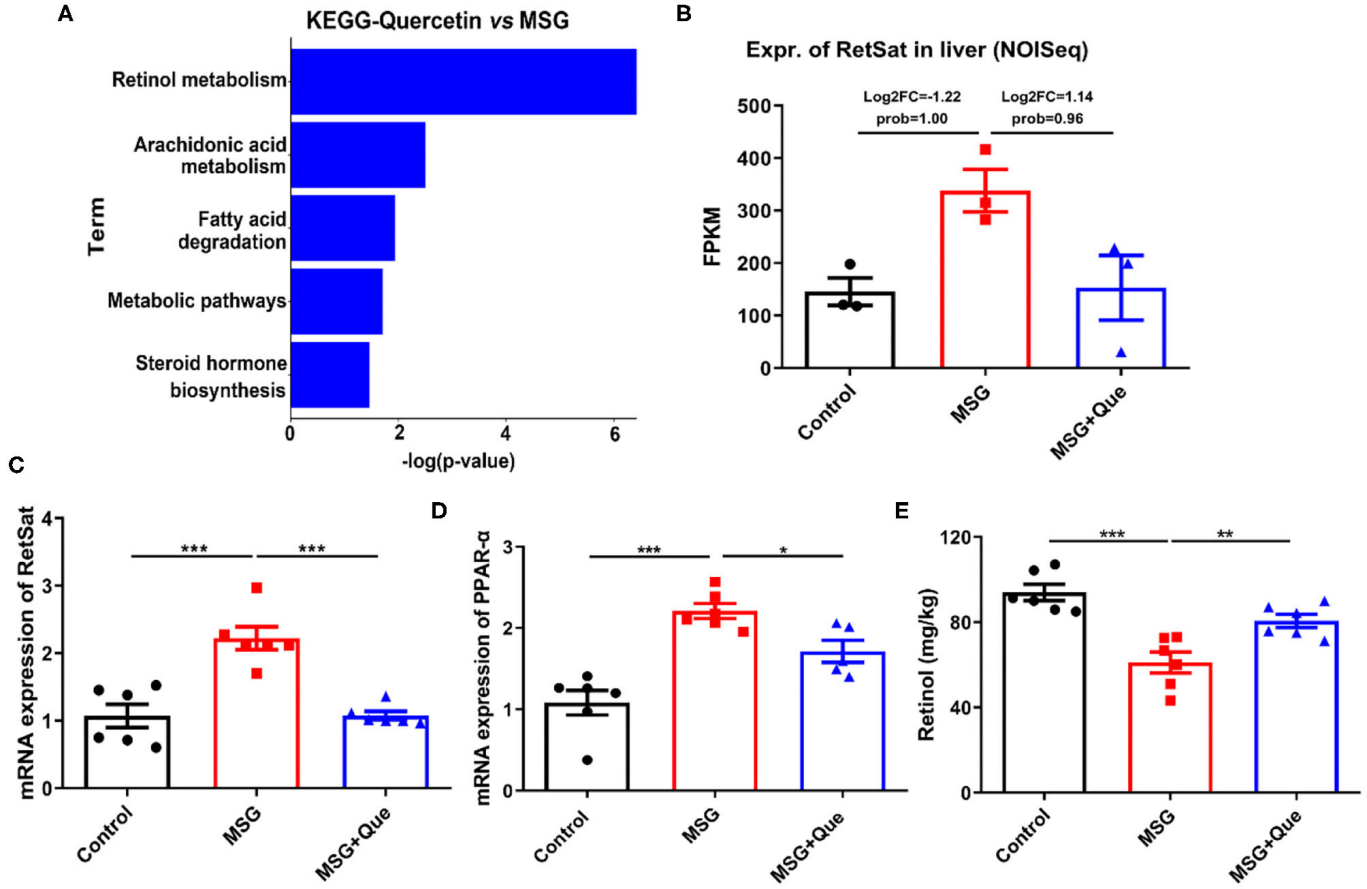
MSG-induced liver RetSat up-regulation is downregulated after the quercetin treatment. **(A)** The functional enrichment of DEGs in the MSG and quercetin-treated group. **(B)** Transcriptome analysis of RetSat in the control, MSG and quercetin-treated group. **(C)** mRNA expression of liver RetSat. **(D)** PPAR-α mRNA level in the liver tissue. **(E)** Retinol content in the liver tissue. FPKM: Fragments per Kilobase Million. Data are presented as mean ± SEM, *n* = 3–6. Data were analyzed by one-way ANOVA followed by *post-hoc* LSD tests. *p < 0.05, **p < 0.01, ***p < 0.001.

Subsequently, to investigate whether microbial structure restored by the quercetin treatment could improve liver metabolism, we also performed transcriptome analysis, and found that the “Retinol metabolism pathway” was mostly affected after the quercetin treatment (Figure 6A). And the RetSat expression level in the liver was remarkably decreased (Figure 6B), which was further confirmed by qRT-PCR (Figure 6C). As expected, *PPAR-*α expression was significantly reduced and retinol content was increased after the quercetin treatment (Figures 6D,E). Therefore, these results demonstrated the quercetin treatment could improve liver lipid metabolism by down-regulating of RetSat expression.

## Discussion

Abdominal obesity is characterized with a heavy load of fatty acids and triglyceride accumulated in liver and other organs, further resulting in organ dysfunction (31). Conventionally, MSG was proved to typically induce abdominal obese mice by hypothalamic damage (6). A latest study showed that enteroendocrine cells synthesize and use glutamate as a neurotransmitter to transduce signals to vagal neurons, so that gut lumen can be rapidly connected to the brainstem (36). Nevertheless, whether gut microbiota play a key role in the MSG-induced hypothalamic damage were not fully explained. In this study, fecal 16S rDNA sequencing results showed gut microbiota composition was dramatically altered in MSG-induced obese mice, whereas obese phenotype was not obvious after microbiota depletion, indicating the crucial roles of gut microbiota in the development of MSG-induced abdominal obesity. Intriguingly, MSG-induced neuronal damage was attenuated after microbiota depletion, while co-housing MSG-treated mice with control mice could lighten neuronal damage, which further suggested that intestinal microbes might participate in the regulation of neuronal damage in abdominal obesity.

In this study, administration of MSG in neonatal mice significantly changed the structure of gut microbiota, characterized with a decrease in abundance of *Bacteroidetes* and an increase in *Firmicutes*, which have been proven to be positively associated with obesity (20). *Bifidobacterium* species were greatly decreased in the MSG-treated group, which was consistent with that in obese individuals (37). At the genus level, decreased abundance of *Bacteroides* and *Akkermansia*, the majority of which could produce anti-inflammation short-chain fatty acids (38), may cause intestinal inflammation and damage gut integrity in abdominal obese mice. Besides, the decrease in relative abundance of *Bacteroides* and *Bifidobacterium* that can degrade MSG in the MSG-treated mice, might increase glutamate accumulation in the body, in agreement with the reports in obese patients (8, 39–41). However, neonatal mouse microbiota alterations in this study were different from those in adult mice induced by MSG as reported (42), which may be ascribed to that infancy is the window period for the formation of stable microbial structures and more susceptible to environmental factors (43).

Interestingly, we discovered that some changes in intestinal bacteria such as *Bacteroidetes* and *Firmicutes* caused by MSG were similar to that induced by high fat diet (HFD) (44). Compared to HFD-induced obesity, the decreased abundance of *Verrucomicrobia* in MSG-induced obese mice may be an important reason for abdominal obesity phenotype. It is noted that gut flora alterations induced by HFD were attributed to high lipid content in the diet, whereas MSG-induced obese mice in our study were fed on a chow diet, which indicated environmental factors that enter the body through other routes besides gastrointestinal route may also induce intestinal dysbiosis. In this study, MSG can induce the gut microbiota dysbiosis via subcutaneous injection, which is suggested that MSG can transfer into blood to alter the gut microbiota after subcutaneous injection, like benzene (45).

Liver lipid metabolism disorder plays an important role in the development of obesity (46). In this study, we performed transcriptome analysis in the liver and revealed retinol metabolism was notably affected in the MSG-treated mice. Retinol is an essential micronutrient for maintaining normal growth and development, barrier integrity, immunity, and vision (47). Retinol precursor carotenoids are absorbed and converted into retinol by enterocytes, and retinol was storage in the liver. The dysregulated retinol metabolism could contribute to disease development, such as non-alcoholic fatty liver disease (NAFLD), steatohepatitis, obesity and insulin resistance (48). Our results showed reduced abundance of *Bacteroides* and *Bifidobacterium* in the MSG-treated mice, which were correlated to facilitate vitamin absorption (49), might account for disordered retinol metabolism in MSG-induced obese mice. In the retinol metabolism pathway, RetSat, a key enzyme coordinates liver lipid metabolism, directly affecting circulating and hepatic triglyceride levels (27), was notably increased in the MSG-treated mice. And its transcriptional regulator PPAR-α was elevated as well. Hepatic PPAR-α level was up-regulated in HFD-induced obesity, which was significantly down-regulated after fecal microbiota transplantation from normal mice. However, the expression of PPAR-α and RetSat had no significant difference between microbiota-depleted control and MSG-treated mice. The results manifested that such microbiota alterations induced by MSG up-regulated PPAR-α mediated RetSat expression, thereby affecting liver lipid metabolism in abdominal obese mice.

As MSG-induced abdominal obesity is serious to human health (50, 51), it is urgent to discover medicines for treating abdominal obesity. Prebiotics and probiotics have the physiologic function of restoring gut microbiota dysbiosis and are beneficial to the body (52, 53). Quercetin, as a prebiotic, has attracted attention for its excellent health benefits, which makes it an important compound for the development of highly effective functional foods (54, 55). Although the quercetin treatment decreased body fat mass, as well as the waist circumference and triglyceride concentration in obese subjects (14), its effects in the MSG-induced abdominal obesity remains unlear. In this study, we found that quercetin treatment protected mice from MSG-induced metabolic disorders. Furthermore, we disclosed that quercetin could improve gut microbiota dysbiosis, down-regulate RetSat expression in the liver, thus preventing MSG-induced abdominal obesity.

## Conclusion

**Figure 7:**
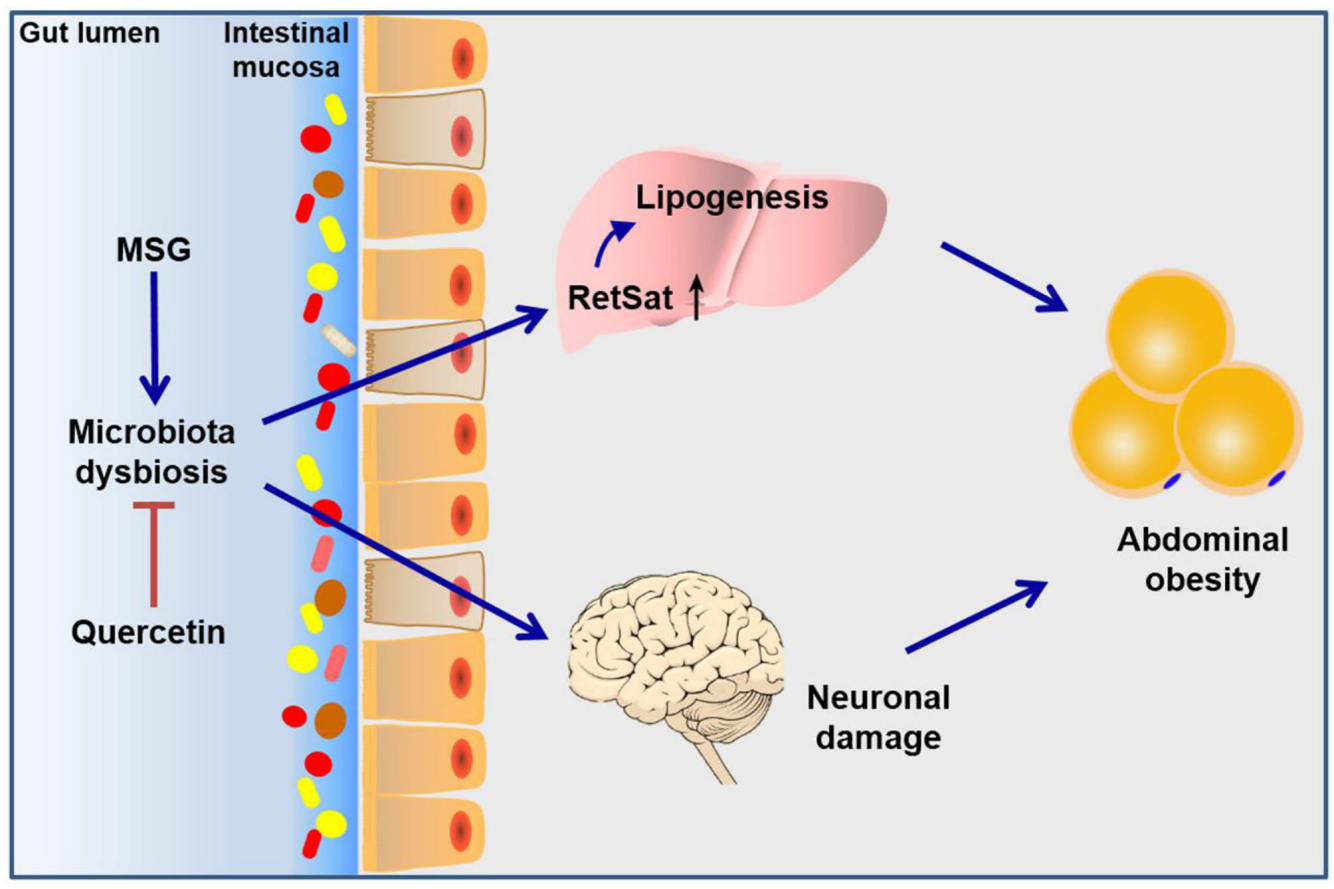
Schematic diagram of the mechanism of MSG-induced abdominal obesity. Our study demonstrated that MSG could cause gut microbiota dysbiosis, leading to up-regulation of RetSat in the liver tissue and neuronal damage in the brain to induce abdominal obesity, which could be ameliorated after quercetin treatment.

In conclusion, this study elucidated the role of gut microbiota dysbiosis in the pathogenesis of MSG-induced abdominal obesity. Such gut microbiota dysbiosis affected retinol metabolism pathway in the liver and neuronal damage in the brain, which jointly resulted in the development of abdominal obesity. What's more, we found quercetin exerted beneficial effects to abdominal obesity through improving microbiota dysbiosis and metabolic disorders (Figure 7). Our study not only enriched the pathogenesis of MSG-induced abdominal obesity, but also established an important role for quercetin in preventing abdominal obesity by modulating gut microbiota.

## Data Availability Statement

The datasets presented in this study can be found in online repositories. The names of the repository/repositories and accession number(s) can be found at: NCBI SRA; PRJNA707230, PRJNA706106, PRJNA706110, PRJNA706138.

## Ethics Statement

The animal study was reviewed and approved by Institutional Animal Care and Use Committee at Tongji Medical College, Huazhong University of Science and Technology (IACUC Number: 2170).

## Author Contributions

YZ, A-YG, and XY designed the experiments. LZ and XZ performed the experiments, analyzed the data, and wrote the draft manuscript. JL assisted the experiments. MX assisted in the analysis of transcriptomics. YZ and A-YG revised and approved the final version. All authors contributed to the article and approved the submitted version.

## Conflict of Interest

The authors declare that the research was conducted in the absence of any commercial or financial relationships that could be construed as a potential conflict of interest.

## Abbreviations

ABX: antibiotic
DEGs: differentially expressed genes
HFD: high fat diet
LEfSe: linear discriminant analysis Effect Size
MSG: monosodium glutamate
NAFLD: non-alcoholic fatty liver disease
OTU: operational taxonomic units
PAS: periodic acid-schiff
PCoA: principal component analysis
PPAR-α: peroxisome proliferator-activated receptor-α
Que: quercetin
RetSat: retinol saturase.
