# Supplementary material for: Quercetin Ameliorates Gut Microbiota Dysbiosis That Drives Hypothalamic Damage and Hepatic Lipogenesis in Monosodium Glutamate-Induced Abdominal Obesity
Source: Front Nutr. 2021 Apr 29;8:671353. doi: 10.3389/fnut.2021.671353 (PMC8116593; doi:10.3389/fnut.2021.671353)
Supplement: Supplementary file 1 [file Data_Sheet_1.docx]

Supplementary Material

Quercetin ameliorates gut microbiota dysbiosis that drives hypothalamic damage and hepatic lipogenesis in monosodium glutamate-induced abdominal obesity

**Lijun Zhao^1#^, Xiaoqiang Zhu^1#^, Mengxuan Xia^2#^, Jing Li^1^, An-Yuan Guo^2*^, Yanhong Zhu^1*^ and Xiangliang Yang^1*^**

^1^ National Engineering Research Center for Nanomedicine, College of Life Science and Technology, Huazhong University of Science and Technology, Wuhan, China;

^2^ Key Laboratory of Molecular Biophysics of the Ministry of Education, Department of Bioinformatics and Systems Biology, College of Life Science and Technology, Huazhong University of Science and Technology, Wuhan, China.

^#^ These authors contributed equally to this work.

***Correspondence:**yhzhu@hust.edu.cn; guoay@hust.edu.cn; nanomedicine@mail.hust.edu.cn

*Address of corresponding authors: No.1037 Luoyu Road, Wuhan, 430074, Hubei, China.

TEL: +86-27-87792147, FAX: +86-27-87792234

**
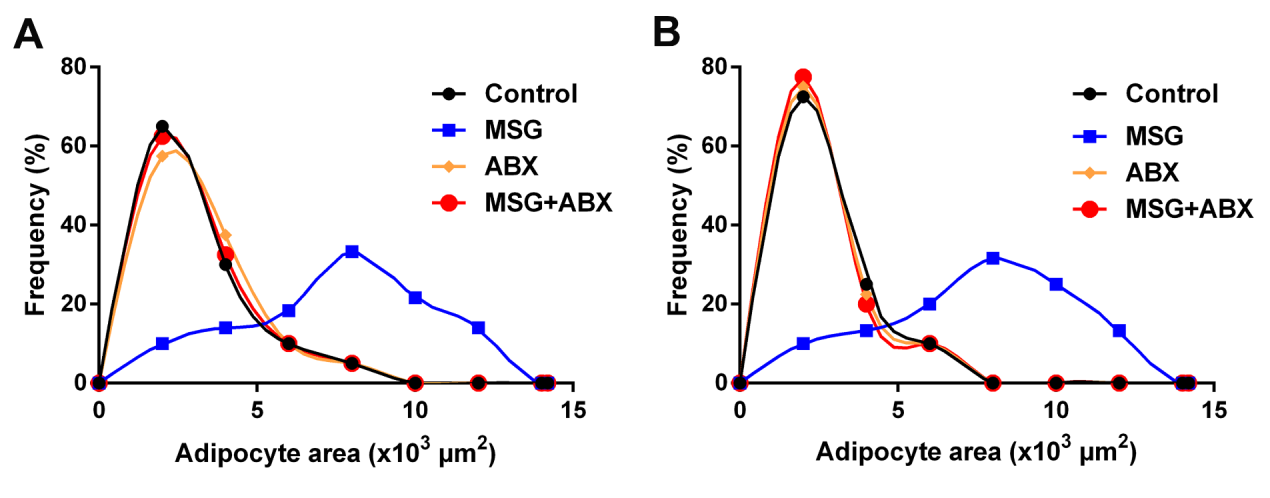
**

**Supplementary Figure 1.** Adipocyte area distribution of **(A)** inguinal and **(B)** mesenteric tissues in control, MSG, ABX and MSG+ABX groups.

**
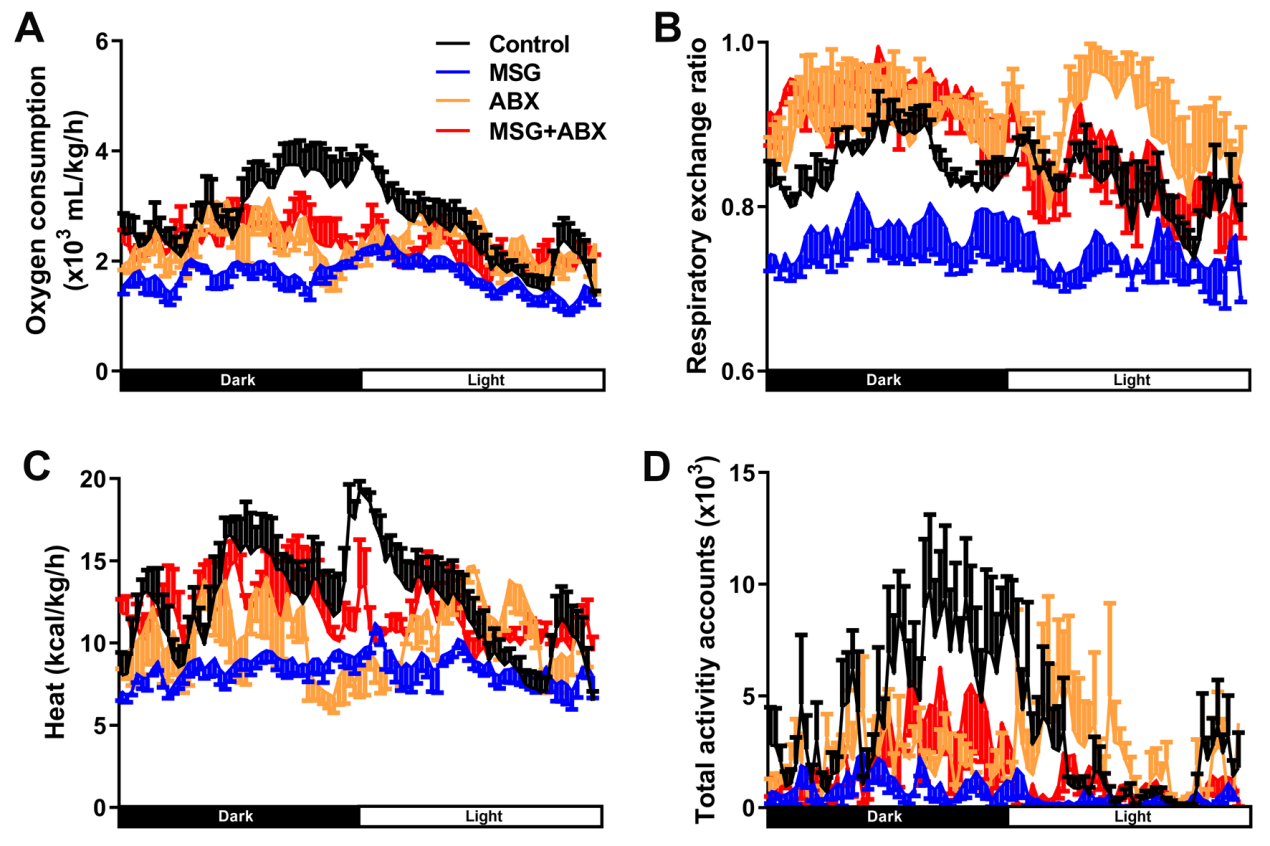
**

**Supplementary Figure 2. Changes of metabolic indexes.** **(A)** Oxygen consumption, **(A)** respiratory exchange ratio, **(B)**heat production and **(D)** total activity accounts over a 24 h period.

**
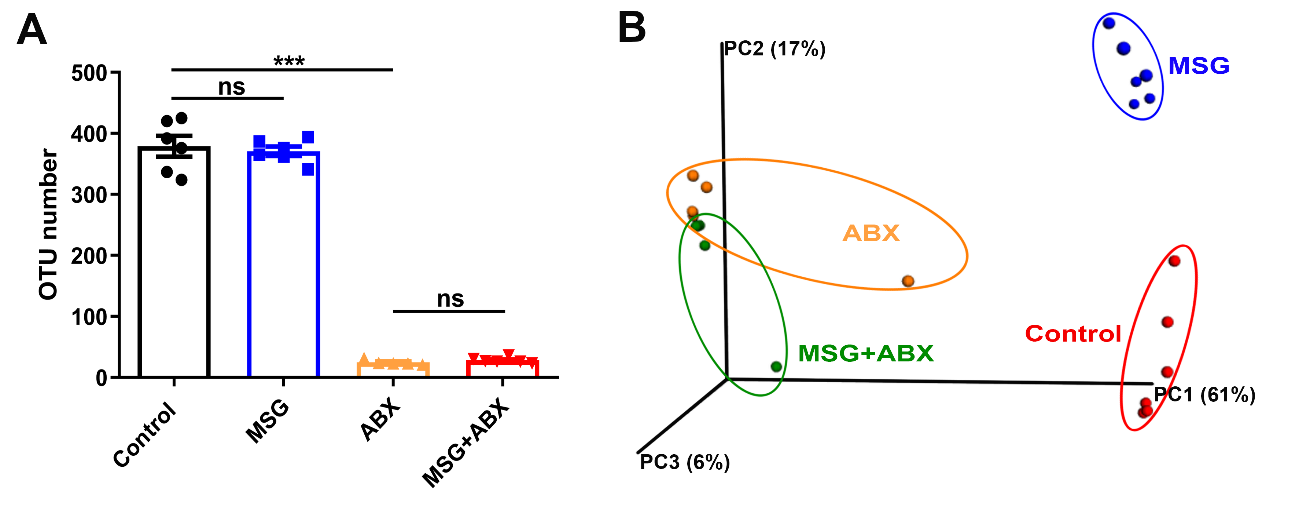
**

**Supplementary Figure 3. 16S rDNA sequencing results.** (**A)** OTU number of mice in the control, MSG, ABX and ABX+MSG group. (**B)** Weighted Unifrac analysis of microbiota. Data are presented as mean ± SEM, n=6, *p< 0.05, **p< 0.01, ***p<0.001, ns, no significance.


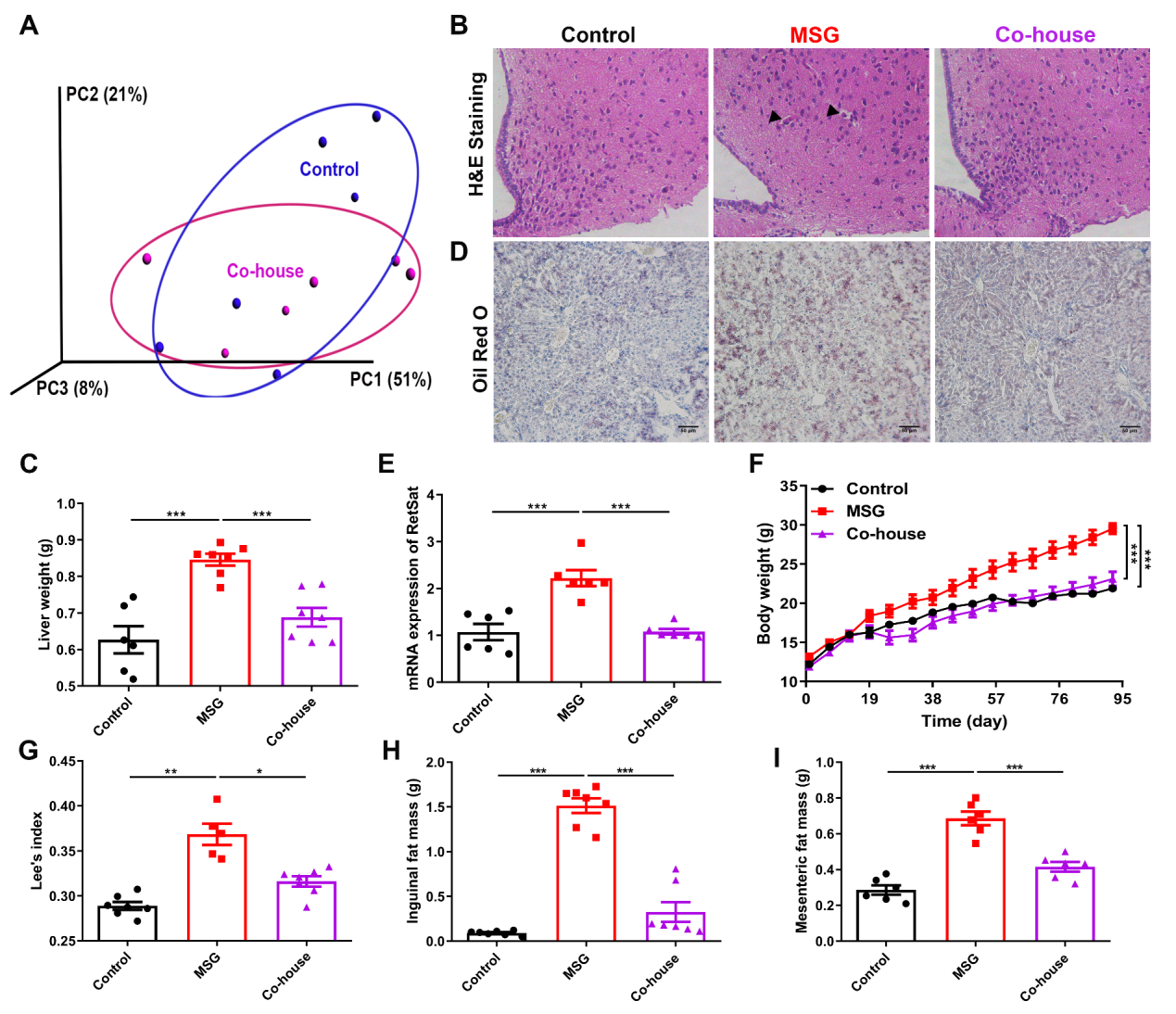


**Supplementary Figure 4. Co-housing alleviates MSG-induced hypothalamic damage and abdominal obesity.** **(A)** Weighted Unifrac analysis of microbiota between the control and co-house group. **(B)** H&E staining in the arcuate nucleus of hypothalamus. **(C)** Liver weight. **(D)** Oil Red O staining of liver tissues. Scale bar: 50 μm. **(E)** Expression levels of RetSat in liver by qRT-PCR. **(F)** Body weight. **(G)** Lee’s index. **(H)** inguinal fat mass. **(I)** Mesenteric fat mass. Data are presented as mean ± SEM, n=5-8, *p< 0.05, **p< 0.01, ***p<0.001. Arrowheads indicate damaged neurons.


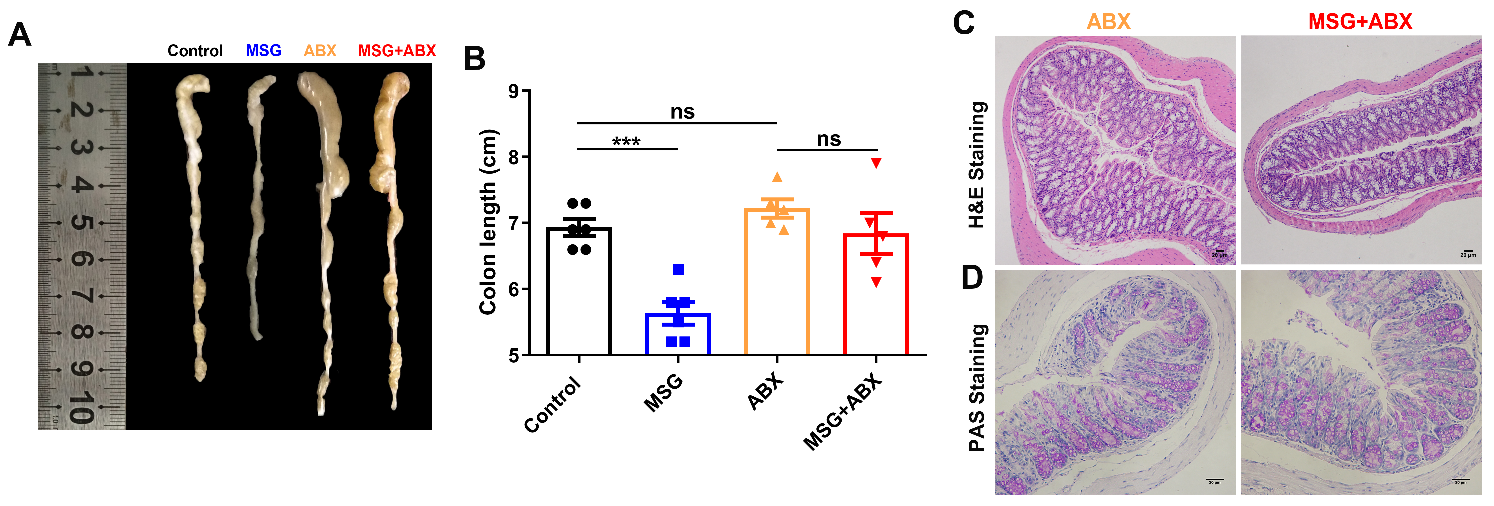


**Supplementary Figure 5. MSG-induced intestinal damage is counteracted by ABX treatment. (A)** Macroscopic changes and **(B)** colon length of mice. **(C)** H&E staining of proximal colon. Scale bar: 20 μm. **(D)** PAS staining of proximal colon. Scale bar: 20 μm. Data are presented as mean ± SEM, n=6, *p< 0.05, **p< 0.01, ***p<0.001, ns, no significance.


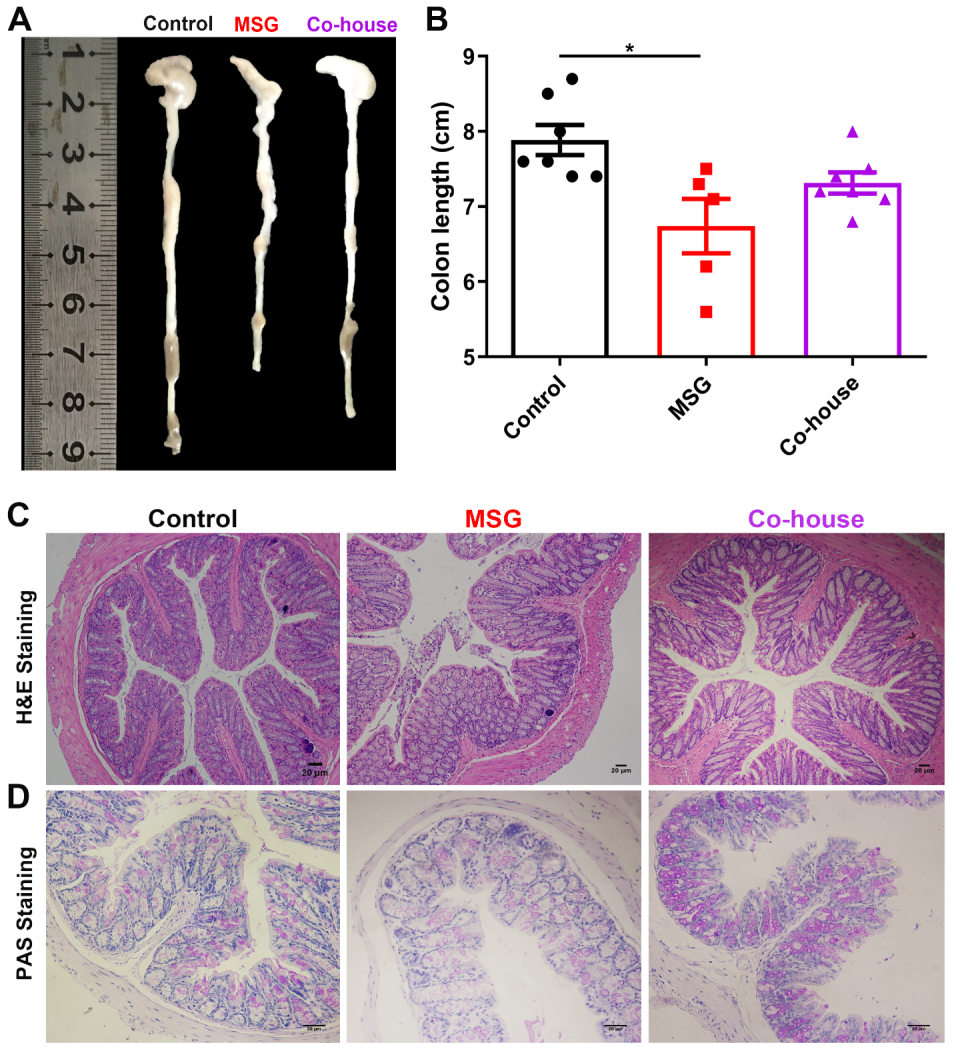


**Supplementary Figure 6. Co-housing alleviates intestinal damage.** **(A)** Corresponding macroscopic changes and **(B)** colon tissue length of the control, MSG and co-house group. **(C)** H&E staining of colon tissues. Scale bar: 20 μm. **(D)** PAS staining of proximal colon. Scale bar: 20 μm. Data are presented as mean ± SEM, n=5-8, *p< 0.05, **p< 0.01, ***p<0.001.


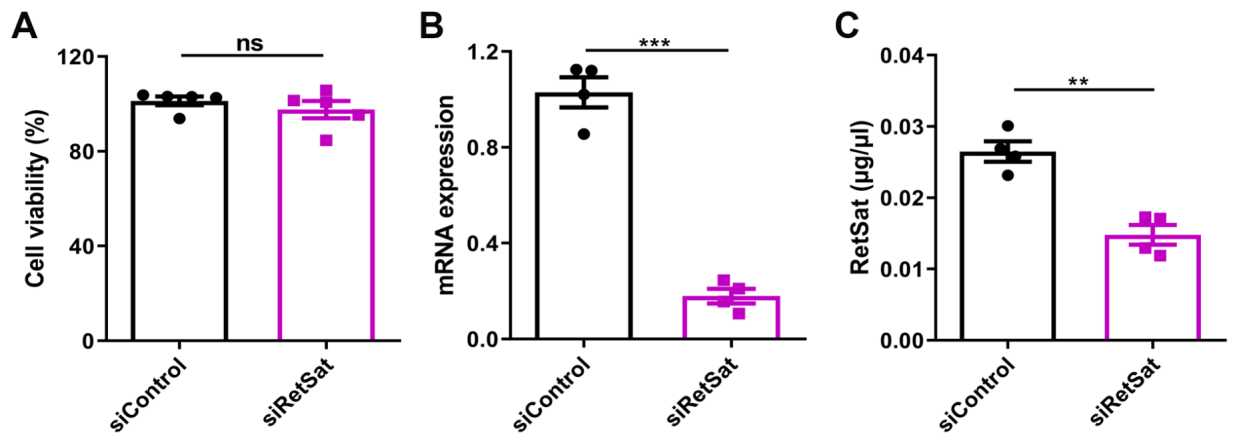


**Supplementary Figure 7. Cell viability and RetSat expression after siRetSat interference.** **(A)** Cell viability after transfection. **(B)** mRNA expression at 24 h and **(C)** protein content at 48 h of RetSat in siControl or siRetSat hepatocytes from MSG-induced obese mice. Data are presented as mean ± SEM, n=4, *p< 0.05, **p< 0.01, ***p<0.001, ns, no significance.


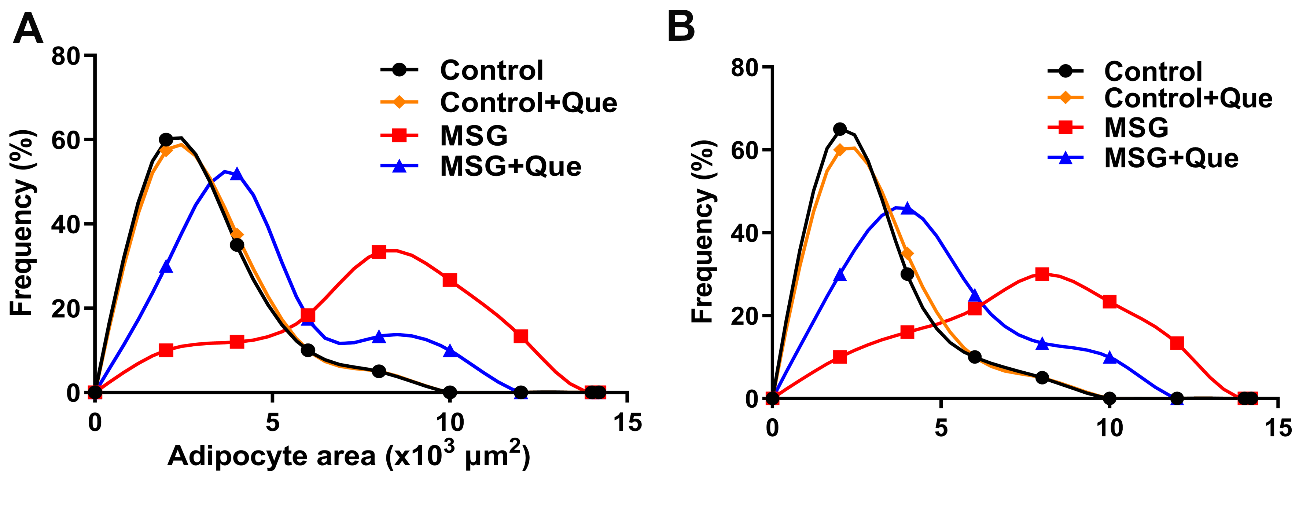


**Supplementary Figure 8.** Adipocyte area distribution of **(A)** inguinal and **(B)** mesenteric tissues in control, control +Que MSG and MSG +Que groups.


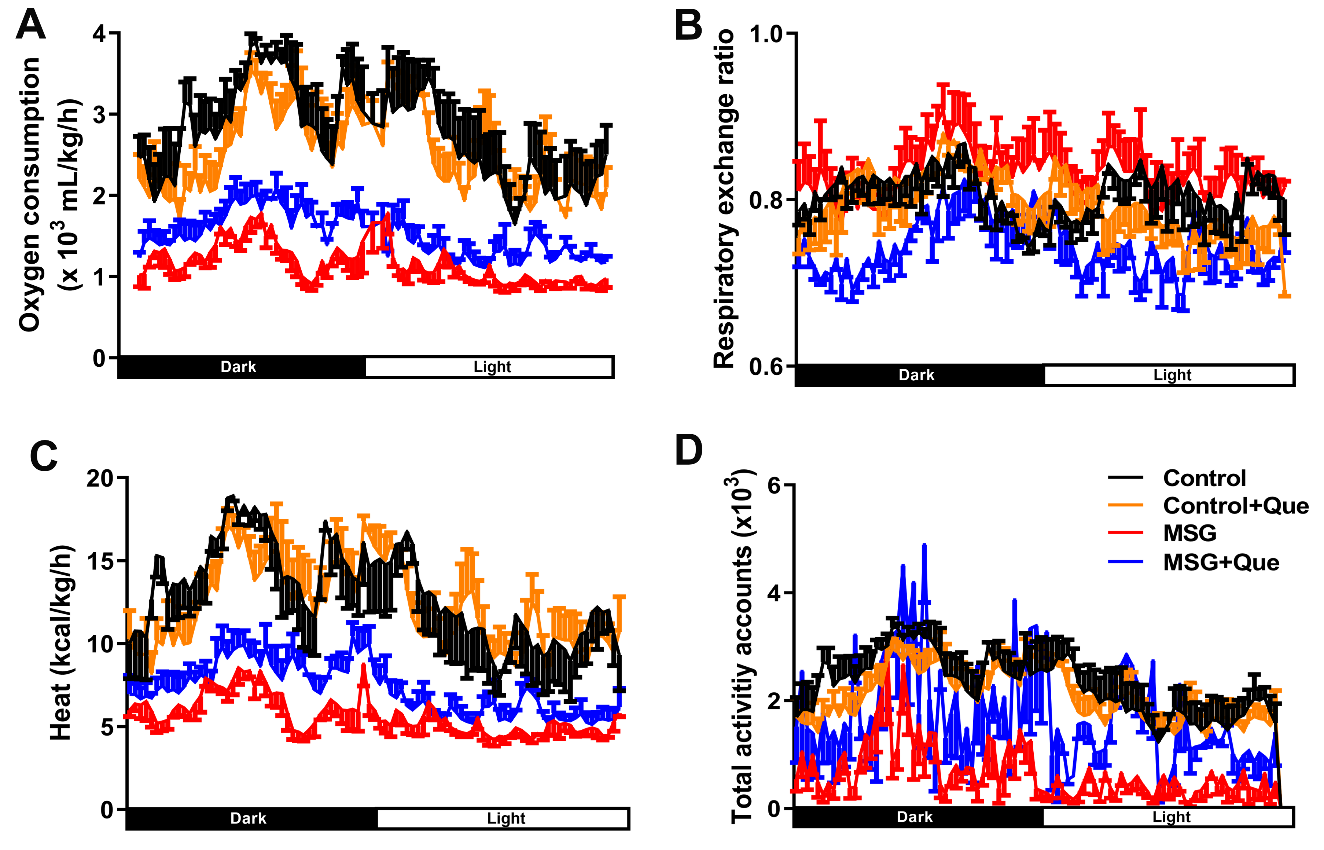


**Supplementary Figure 9. Changes of metabolic indexes after quercetin treatment. (A)** Oxygen consumption, **(B)** respiratory exchange ratio, **(C)** heat production and **(D)** total activity accounts over a 24 h period.

**Supplementary Table 1. Nucleotide sequences used in this study**

| qPCR | mChrebp fw | CGGGACATGTTTGATGACTATGTC |
| --- | --- | --- |
|  | mChrebp rv | CATCCCATTGAAGGATTCAAATAAA |
|  | mFasn fw | GGAGGTGGTGATAGCCGGTAT |
|  | mFasn rv | TGGGTAATCCATAGAGCCCAG |
|  | mMuc2 fw | ATGCCCACCTCCTCAAAGAC |
|  | mMuc2 rv | GTAGTTTCCGTTGGAACAGTGAA |
|  | mPpar-α fw | AGAGCCCCATCTGTCCTCTC |
|  | mPpar-α rv | ACTGGTAGTCTGCAAAACCAAA |
|  | mReg3γ fw | ATGCTTCCCCGTATAACCATCA |
|  | mReg3γ rv | GGCCATATCTGCATCATACCAG |
|  | mRetSat fw | GTCTACGTGGGCCTTTACGC |
|  | mRetSat rv | ACTTTCTTCCTAGCCTCCTTGTC |
|  | mZO-1 fw | CTCCAGGTGCTTCTCTTGCT |
|  | mZO-1 rv | TATCTTCGGGTGGCTTCACT |
|  | mβ-actin fw | GGCTGTATTCCCCTCCATCG |
|  | mβ-actin rv | CCAGTTGGTAACAATGCCATGT |
| siRNA | Control siRNA-sense | CACUCAAGAUUGUCAGCAATT |
|  | Control siRNA-antisense | UUGCUGACAAUCUUGAGUGAG |
|  | RetSat siRNA-sense | GCGGCUGUUGUCAUACCUUTT |
|  | RetSat siRNA-antisense | AAGGUAUGACAACAGCCGCTT |
